# Parasitoids and Predators of the Invasive Aphid *Melanaphis sorghi* Found in Sorghum and Non-Crop Vegetation of the Sorghum Agroecosystem

**DOI:** 10.3390/insects13070606

**Published:** 2022-07-06

**Authors:** Ashleigh M. Faris, Michael J. Brewer, Norman C. Elliott

**Affiliations:** 1 Department of Entomology, Texas A&M University, TAMU 2475, College Station, TX 77843, USA; mjbrewer@ag.tamu.edu; 2 Department of Entomology, Texas A&M AgriLife Research & Extension Center, 10345 State Hwy 44, Corpus Christi, TX 78406, USA; 3 United States Department of Agriculture—Agricultural Research Services, 1301 North Western Road, Stillwater, OK 74075, USA; norman.elliott@usda.gov

**Keywords:** Aphididae, Apehlinidae, Coccinellidae, natural enemies, *Melanaphis sorghi*

## Abstract

**Simple Summary:**

The sorghum aphid is an invasive pest of grain sorghum in North America; their infestations when in high numbers can reduce grain sorghum yield. Fortunately, there are numerous beneficial insects such as parasitoid wasps, lady beetles, hoverfly and lacewing larvae that will feed on these aphids. These beneficial insects are naturally occurring in local habitats such as grasses and shrubs, Johnson grass, and cropland surrounding grain sorghum during and after sorghum production. The goal of this study was to estimate the relative effect of these habitats to serve as a source of natural enemies of the sorghum aphid in- and off-season of sorghum production. This study was conducted over two years and the results found that predators (lady beetles and their larvae, hoverfly and lacewing larvae) were most diverse in the habitat containing grasses and shrubs and most abundant during the sorghum-growing season. Parasitoid wasps were abundant across all habitat types during and outside of the sorghum-growing season. These results highlight the potential importance of persistence of natural enemies across vegetation types associated with their ability to manage sorghum aphid infestations. The natural enemies in these habitats are well positioned to play a role in suppressing sorghum aphid.

**Abstract:**

*Melanaphis sorghi* (Theobald) (sorghum aphid), (=*Melanaphis sacchari* Zehntner) (Hemiptera: Aphididae), is an invasive pest of *Sorghum bicolor* (L.) in North America. Over 19 species of predators and parasitoids have been found to prey on *M. sorghi*. Natural enemies may reside in vegetation such as sorghum in cultivation (in-season) and persist after harvest (off-season), in Johnson grass (*Sorghum halepense*) (L.) and riparian areas consisting of shrubs and grasses, including Johnson grass. The objective was to assess the ability of these vegetation types to harbor *M. sorghi* natural enemies during and between annual grain sorghum production. Predator diversity was greatest in riparian vegetation in-season, with twelve species detected across seven families, and four orders of insects. Six lady beetle (Coleoptera: Coccinellidae) species were abundant in-season, and *Cycloneda sanguinea* (L.) persisted at relatively high abundance off-season. Parasitoid diversity was more limited (two primary parasitoids and one hyperparasitoid detected) with the primary parasitoids commonly detected. *Aphelinus nigritus* (Howard) (Hymenoptera: Aphelinidae), accounted for 85% and 57% of parasitoids in- and off-season, respectively. *Aphelinus nigritus* abundance was steady across the annual sorghum season in all vegetation types. Results from this study will inform land-management strategies on how diverse vegetations can play a role in the biological control of *M. sorghi.*

## 1. Introduction

*Melanaphis sorghi* (Theobald) (sorghum aphid), (previously described as =*Melanaphis sacchari* Zehntner) (Hemiptera: Aphididae) is an invasive pest of sorghum, *Sorghum bicolor* (L.), in North America. Since its first detection on sorghum along the Texas Gulf Coast and Louisiana in 2013, *M. sorghi* has spread to 17 states in the United States of America (U.S.) and sorghum-producing areas in Mexico and Caribbean islands. This aphid was not previously considered a significant sorghum pest in North America [1]. Supported by morphometric and molecular research, the aphid on sorghum and Johnson grass is considered a superclone, distinct from that found on sugarcane [2]. It was reclassified as *Melanaphis sorghi* (Theobald) (Hemiptera: Aphididae) with its likely origin in Africa or Asia [3]. Since 2013, *M. sorghi* outbreaks on sorghum have caused economic loss resulting from direct plant damage that reduces seed yield and from indirect seed yield loss due to aphid honeydew disrupting mechanical harvest [1]. In response, economic thresholds and sampling protocols were established to guide the use of insecticides [4,5,6], and sorghum hybrids partially resistant to the aphid were identified [7].

Natural enemies of *M. sorghi* have been detected from Mexico to Kansas, U.S., consisting of predators and parasitoids [8,9,10,11], and this complex is similar to that seen preying on other cereal aphids in the North American Great Plains [12]. From south to central Texas, where about 15% of U.S. sorghum is grown, and where this study is located, predators include nine lady beetle species (Coleoptera: Coccinellidae), brown lacewings (Neuroptera: Hemerobiidae), five green lacewing species (Neuroptera: Chrysopidae), three hoverfly species (Diptera: Syrphidae), and one minute pirate bug (Hemiptera: Anthocoridae) [11]. This natural enemy complex is generally similar to, but with more species than, that seen in Kansas [8]. The primary parasitoids detected were the endoparasitoids *Aphelinus nigritus* Howard (Hymenoptera: Aphelinidae) and *Lysiphlebus testaceipes* (Cresson) (Hymenoptera: Braconidae), which produce black-blue and brown mummified aphids, respectively [1,11]. *Syrphophagus aphidivorus* (Mayr) (Hymenoptera: Encyrtidae) has also been detected functioning as a hyperparasitoid [11]. In sorghum production regions in northeastern Mexico, within 300 km of the area of this study, the same primary parasitoids were detected as well as *Aphidius* sp. (Hymenoptera: Braconidae). A complex of coccinellid, syrphid, and lacewing predators is also similar [9].

*Melanaphis sorghi* survives mild winters in the southern latitudes of North America, such as southern Texas and Mexico, on ratoon and regrowth sorghum and Johnson grass, *Sorghum halepense* (L.) (Poales: Poaceae) [1]. Annually planted sorghum may be infested through wind-aided movement of sugarcane alates derived from these local sources or sources further south. *Melanaphis sorghi* can spread annually into northern temperate regions where its persistence on sorghum regrowth and Johnson grass is likely much lower during the colder winters. Long-distance, wind-aided movement of alates from maturing sorghum in the south to northern regions appears to be an important mechanism of the annual northerly spread [13]. This capacity to rapidly spread to annually cultivated grain sorghum fields, and the aphid’s high reproductive growth rate, can result in a short period of about one month between initial aphid infestation and sorghum injury [4]. This window of risk is affected by the timing of aphid migration [13], the aphid reproduction rate as moderated by environmental conditions [4], and the susceptibility of the sorghum colonized [5]. These attributes provide a challenge to *M. sorghi* population regulation by natural enemies.

Persistence of aphid natural enemies within non-crop vegetation growing in the vicinity of sorghum and on sorghum regrowth may be relevant to the ability of natural enemies to respond to *M. sorghi* infestations on sorghum as is seen with other cereal aphids. In the southern Great Plains, Michels and Matis [14] reported that corn leaf aphid, *Rhopalosiphum padi* (L.) (Hemiptera: Aphididae), was an effective early-season aphid host of *L. testaceipes* on sorghum and winter wheat. Injury caused by corn leaf aphid was negligible. The parasitoid increased in abundance by preying on corn leaf aphid, which led to increased subsequent biological control of greenbug, *Sitobion avenae* (F.) (Hemiptera: Aphididae), and decreased greenbug-induced injury to sorghum. In Europe, aphid parasitoid abundance increased in winter wheat planted in a patchy matrix of woodlands, grasslands, and croplands, compared to areas of more concentrated cereal production. Unfortunately, the benefits of biocontrol were not realized because aphid abundance on the cereal crop also increased in the more diverse landscapes [15].

Specific to the sorghum agroecosystem, seasonal occurrence and composition of non-crop vegetation are attributes that may be relevant to natural enemies suppressing *M. sorghi* in the protected sorghum crop. For example, Johnson grass, adjacent to sorghum fields in riparian areas and agricultural ditches, may provide green vegetation for *M. sorghi* [2] and other aphids that support natural enemy persistence, especially when sorghum is not in cultivation. Sorghum regrowth after grain harvest that persists through mild winters in southern regions may serve the same function (A.M.F., M.J.B. personal observation). The objective of this study was to evaluate the ability of different vegetation types to harbor parasitoids and predators of *M. sorghi*. The hypothesis was that non-crop vegetation harbors natural enemies of *M. sorghi* during and outside of the period of annual sorghum cultivation. These data contribute to the discussion of whether selected non-crop vegetation may serve as indicators of top-down regulatory quality of the sorghum agroecosystem in southern latitudes where *M. sorghi* persists year-round.

## 2. Materials and Methods

### 2.1. Natural Enemy Seasonal Assessment in Three Vegetation Types

Parasitoids and predators were identified, and relative abundance was measured from recoveries obtained from *M. sorghi*-infested sorghum planted in pots. The pots were placed in three vegetation types: sorghum in cultivation (in-season) and regrowth sorghum after harvest (off-season), relatively pure Johnson grass patches, and riparian areas that included Johnson grass. Sets of pots were placed in the three vegetation types at multiple time periods when sorghum was in cultivation (in-season) and after grain sorghum had been harvested and prior to planting seed in the spring (off-season). This method has previously been used to survey the activity of natural enemies of aphids in other cereal-based agroecosystems [16,17,18].

Round plastic pots (15.24 cm height, 16.83 cm diameter) that contained multiple 3–4 leaf stage sorghum plants were infested with ca. 500 *M. sorghi* from a laboratory colony free of natural enemies. The colony was maintained on a grain sorghum hybrid known to be susceptible to *M. sorghi* (DKS 3888, Bayer Inc., St. Louis, MO, USA) [19]. The colony was originally established from, and was periodically supplemented with, field-collected *M. sorghi* taken from sorghum growing where the current study was conducted in Corpus Christi, TX. Before placement in the field, aphids were allowed to reproduce and acclimate on the potted sorghum plants in the greenhouse for five to seven days. The pots were placed in screen cages (ca. 0.5 mm mesh fabric, BioQuip, Rancho Dominguez, CA, USA) designed to exclude small insects, including aphid parasitoids and predators. When the pots were ready for field placement, three pots were kept in the fine mesh cages within the greenhouse to check for parasitoid and predator contamination in the pots. No contamination was detected during the two-year study (data not shown).

Pots of the aphid-infested sorghum were randomly placed along the edge of a 50 ha experimental farm where the three vegetation types were available: a riparian area including Johnson grass and a relatively pure small plantings (ca. 10 m^2^ patches) of Johnson grass plantings ran parallel to the sorghum fields. The pots were placed at least 5 m into a vegetation type. The pots were sunk to about 4 cm into the soil or secured with dense ground vegetation to maintain stability and soil moisture. *Melanaphis sorghi* on the plants were exposed to parasitoids and predators for three or four days with the retrieval period spread equally across each vegetative type (i.e., this facilitated efficient data collection without introducing bias across the treatments). The process was repeated at four time-periods within the sorghum growing season (in-season) from April to September 2018 and 2019. The in-season time periods were (i) after sorghum was planted but before emergence, (ii) during sorghum vegetative growth at about five true leaves (growth stage 4 [20]), (iii) during sorghum flowering (growth stage 10), and (iv) when sorghum-head development reached hard dough (growth stage 11.3). The process was again repeated at three off-season time periods when sorghum was not in cultivation from October 2018 to March 2019, and once in December 2019. Data from collections taken from sorghum at hard dough for the 2019 season were not available for analysis because unusually high greenhouse temperatures (>37 °C) resulted in aphid decline on pots infested in the greenhouse. Ten pots (replications) were set out in each vegetation type and time period during the two-year study.

At the end of the aphid exposure period, ladybugs, lacewings, true bugs, and predatory flies were immediately removed by hand from the pots directly in the field. Two experienced samplers worked side by side throughout the experiment to reduce sampling variation. Adults and immatures of these natural enemies were stored in 70% ethanol for species identification. For parasitoid recovery, pots were placed back into fine mesh cages in the greenhouse for one week. Plants were then clipped and placed into round emergence canisters measuring 0.127 m in diameter and 0.61 m in length. They were made from pressed cardboard with an inverted white funnel and a clear plastic vial attached to one end to collect emerging parasitoids attracted to the light [17]. After two weeks to allow immature parasitoids to complete development, emerged parasitoids were collected from the vials, and the contents of the emergence canister were thoroughly inspected to collect remaining parasitoids. Recovered parasitoids were sorted and counted by morpho-species and stored dry in a glass vial for species-level identification.

### 2.2. Natural Enemy Species Identification and Data Analysis

Natural-enemy species were identified using the Gordon [21] and Havelka et al. [22] keys, and the identification of representative specimens was verified by experts (see acknowledgements). Natural-enemy abundance data were recorded by species, vegetation type, time period, and year. The abundance of each species was also summed across the three vegetation types during the in-season and off-season time periods for the two years of the study to obtain an overview of relative abundance among species. The hypothesis of equality of proportions across species was tested separately for the in-season and off-season periods with the χ^2^ goodness of fit test [23].

Year to year shifts in occurrence and abundance of some species were observed. Therefore, for each year the most frequently occurring predators and parasitoids were selected to test for differences in each species’ abundance across the three vegetation types and across in-season and off-season time periods. In an analysis of variance for each year (Proc GLM, [24]), vegetation type was the main factor, and time period was treated as a split plot factor. Time period was considered a split factor because new pots with aphid-infested plants were placed in the three vegetation types at each time period. In relation to the split plot in time design, the error terms for vegetation type and time period were their respective interactions with replication, while the model residual was used as the error term for the vegetation type by time period interaction [25]. Data transformation for the natural enemy count-based data with zeroes (square root of the value + 0.5) was performed before analyses, as standard practice to compensate for potential variation from normality [25]. If a vegetation type by time period interaction was detected, the model was sliced by time period and Tukey’s means separation test was used to detect differences across vegetation means at each time period [24]. If the interaction was not significant, Tukey’s means separation test was applied to the vegetation type and time period factors [25].

### 2.3. Background M. sorghi Densities

Density estimates of *M. sorghi* were taken periodically during in-season and off-season periods of this study. In-season, *M. sorghi* data were taken in the same sorghum fields used in the experiment. Multiple time points of *M. sorghi* sampling overlapped with the time periods of the experiment. Off-season, *M. sorghi* data were collected from remnant sorghum in the same fields and Johnson grass plantings. Sampling was undertaken across multiple time points from sorghum harvest until sorghum planting in the next growing season. *Melanaphis sorghi* data were collected by randomly inspecting a top and bottom leaf for *M. sorghi* on thirty sorghum plants and ten Johnson grass plants. Aphids were counted and averaged per leaf.

## 3. Results and Discussion

Two species of primary parasitoids were the most abundant natural enemies collected, followed by six species and morphospecies of lady beetles (63% and 36% of 820 recoveries of natural enemies, respectively). The abundance of the parasitoids *A. nigritus* and *L. testaceipes*, and the predators *C. sanguinea*, *Scymnus* spp., and *C. septempunctata*, indicated that they were key natural enemies of *M. sorghi* in this sorghum agroecosystem, represented in these three vegetation types. They had disproportionately high rates of recovery in-season (χ^2^ = 142; df = 11; *p ≤* 0.0001), off-season (χ^2^ = 177; df = 11; *p* ≤ 0.0001), or both (Table 1). Of the primary parasitoids, proportionately more *A. nigritus* were found in-season than off-season, while representation of *L. testaceipes* was more common off-season (Table 1). Hyperparasitism by *S. aphidivorus* was observed in less than 2% of the emerged parasitoid samples in- and off-season (Table 1). Lady beetle adults were well represented in the recoveries. *Cycloneda sanguinea* (L.), *Scymnus* spp., and *Coccinella septempunctata* (L.) (Coleoptera: Coccinellidae) were similar in abundance in-season, while *C. sanguinea* was the dominant species recovered off-season (Table 1). Lady beetle larvae, syrphid larvae, and lacewing larvae made up less than 1% of the total predators collected in-season and off-season; therefore, larval identification was limited to family-level (Table 1). Parasitoids *A. nigritus* and *L. testaceipes* and predators *C. sanguinea*, *Scymnus* spp., and *C. septempunctata* were key members by abundance, based on the equality test of proportions. They each represented greater than 10% of the natural enemies in the parasitoid and predator collections (χ^2^ = 306.31; df = 11; *p* = < 0.0001) (Table 1).

### 3.1. Parasitoids Found in Three Vegetative Types

**Figure 1:**
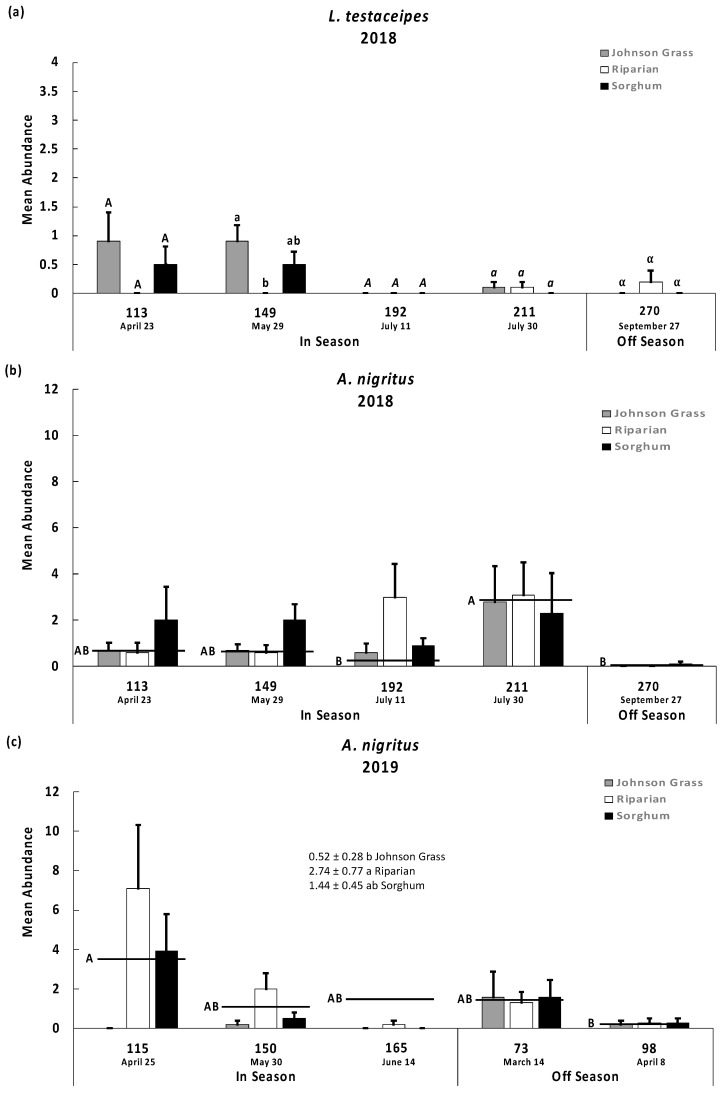
Recoveries (mean abundance per pot are bars and SEM are lines extending from bars) of two parasitoids: (**a**) *Lysiphlebus testaceipes* in 2018, (**b**) *Aphelinus nigritus* in 2018, and (**c**) *A. nigritus* in 2019) in three habitat sites (Johnson Grass pure stand, Riparian area including Johnson Grass, and Sorghum field) during selected dates when sorghum was in cultivation (In Season) and out of cultivation (Off Season). Recoveries were taken from pots planted with sorghum and infested with *M. sorghi*. For. *L. testaceipes* in 2018, means among the three habitats were compared separately for each date of collection. For *A. nigritus* in 2018, means for each date were averaged across the habitats and compared based on ANOVA results presented in text. For *A. nigritus* in 2019, means for each habitat were averaged across the dates of collection and compared. Tukey’s means separation test was used for the comparisons. Means sharing a common symbol (separate comparisons per the ANOVA were indicated by uppercase, lowercase, italics, and Greek lettering) did not significantly differ. Comparisons for *L. testaceipes* were not conducted in 2019 due to lack of significant ANOVA results and low recovery. Please note the change in scale for the y-axis.

A significant vegetation type by time period interaction was detected for *L. testaceipes* (F = 2.39; d.f. = 8, 135; *p* = 0.02) in 2018 (Figure 1a, note change in y-axis scale), while the species was not detected in 2019. In the April and May sampling times, *L. testaceipes* was primarily detected in Johnson grass and sorghum, and seldom recovered in the riparian area (Figure 1a). During the rest of the in-season dates and in September after harvest, it was relatively low in abundance (Figure 1a). The spring period of *L. testaceipes* abundance was within one month of the sorghum planting period in south Texas. Although *L. testaceipes* is typically low in abundance, its periodic population increase and presence off-season in Johnson grass and remnant sorghum provide opportunity for parasitoid respond to *M. sorghi* outbreaks.

Variation across time period (but not vegetation type) was detected for *A. nigritus* (F = 7.50; d.f. = 4, 8; *p* = <0.0001) and the interaction was not significant *(p* > 0.05) in 2018 (Figure 1b). In 2019, *A. nigritus* was again common, and variation was detected across time period (F = 3.32; d.f. = 4, 8; *p* = 0.0125) and vegetation type (F = 6.40; d.f. = 2, 8; *p* = 0.0022) (Figure 1c). The interaction was not significant *(p* > 0.05) in either year (Figure 1b,c). *Aphelinus nigritus* abundance was relatively high across the three vegetation types, just preceding harvest (2018) (Figure 1b) and during the spring before planting (2019) (Figure 1c). Averaging across all time periods, *A. nigritus* was least abundant in Johnson grass. The species was readily detected in both sorghum and the riparian area (Figure 1b,c). Its abundance across the three vegetation types and its representation in-season and off-season bodes well for its function as a key suppression agent for *M. sorghi*. *Aphelinus nigritus* has been found consistently from south to central Texas on both susceptible and aphid-resistant sorghum [11,26,27]. *Aphelinus* sp. (species not identified) has also been reported in northeastern Mexico [28], which may be the same species as in the current study. *Aphelinus* sp. suppressed *M. sorghi* placed on potted sorghum set in a sorghum field as well as the edge of a wooded area [8]. Another species, *Aphelinus albipodus* Mordvilko (Hymenoptera: Aphelinidae) suppressed Russian wheat aphid on wheat in the northcentral U.S. Great Plains. Furthermore, additional in-crop and off-crop vegetation-aided parasitism, when observing parasitism by *A. albipodus* on Russian wheat aphid, on wheat placed in a strip-rotation with sunflower and when the crops were embedded in a more plant-diverse area of the landscape [17,29].

*Syrphophagus aphidivorus* hyperparasitism was restricted to *A. nigritus*, and its abundance was low in this study (<2% of the recoveries, Table 1). In contrast, Maxson et al. [11] reported a hyperparasitism rate of 90% in a central Texas location when *A. nigritus* was at peak abundance, which poses a question about the regulatory potential of *A. nigritus*. Our study supports that *A. nigritus* persists in cultivated sorghum in-season and remnant sorghum off-season, as well as riparian vegetation next to sorghum plantings. Based on *A. nigritus* abundance in the spring before sorghum planting and early season activity in cultivated sorghum, hyperparasitism appears less of a concern during the month-long window when *M. sorghi* first infests sorghum fields in production.

*Lysiphlebus testaceipes* is a known biological control agent for aphids and other pests in a variety of crops [30,31]. It is the dominant parasitoid of greenbug on wheat in the Great Plains in Oklahoma and has also been detected preying on other cereal aphids in the Great Plains [18,32]. It has been detected preying on sugarcane aphid in Mexico [9,10,27,28]; however, its abundance in south Texas has been variable over the last several years (A.M.F., M.J.B. personal observation). Maxson et al. [11] reported *A. nigritus* as the most abundant and consistently occurring parasitoid, while *L. testaceipes* was present in lower abundance with episodes of high parasitism in south and central Texas ([11], M.J.B., A.M.F. personal observation).

### 3.2. Predators Found in Three Vegetative Types

**Figure 2:**
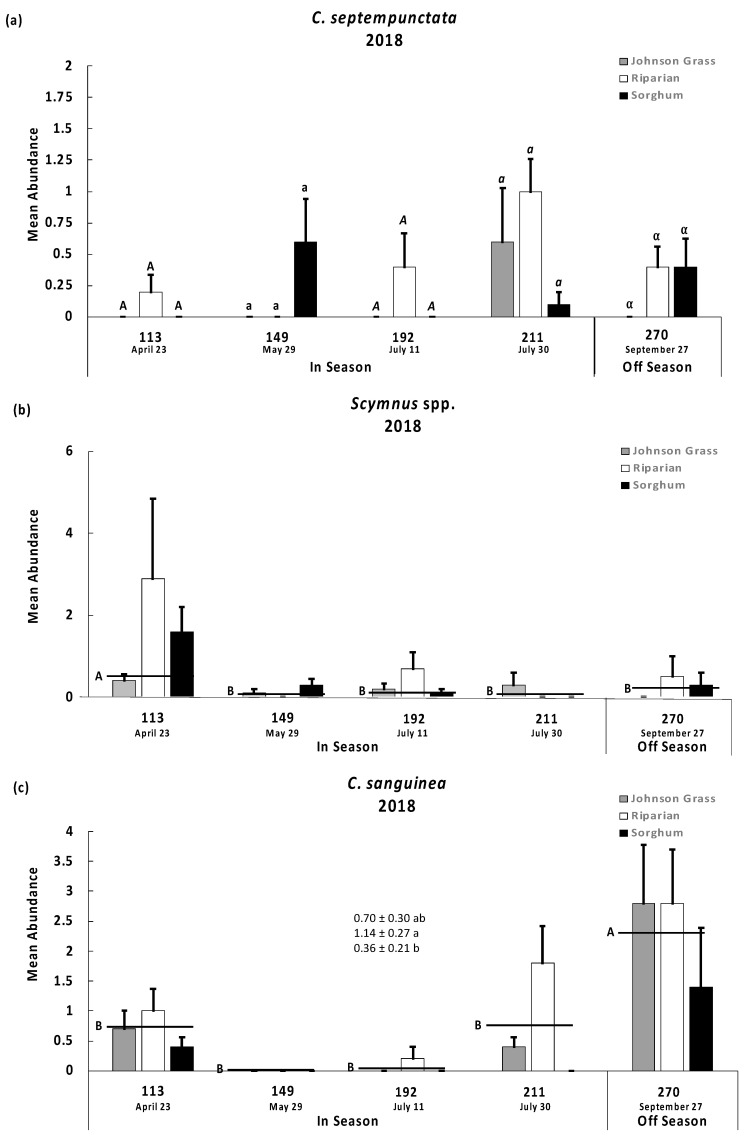
Recoveries (mean abundance per pot are bars and SEM are lines extending from bars) of three predators: (**a**) *Coccinella septempunctata*, (**b**) *Scymnus* spp., and (**c**) *Cycloneda sanguinea*, in three habitat sites (Johnson Grass pure stand, Riparian area including Johnson Grass, and Sorghum field) during selected dates when sorghum was in cultivation (In Season) and out of cultivation (Off Season). Recoveries were taken from pots planted with sorghum and infested with *M. sorghi*. For *C. septempunctata* in 2018, means among the three habitats were compared separately for each date of collection. For *Scymnus* spp. in 2018, means for each date were averaged across the habitats and compared. For *C. sanguinea* in 2018, means for each habitat were averaged across the dates of collection and compared based on ANOVA results presented in text. Tukey’s means separation test was used for the comparisons. Means sharing a common symbol (separate comparisons per the ANOVA were indicated by uppercase, lowercase, italics, and Greek lettering) did not significantly differ. Comparisons for these predators were not conducted in 2019 due to lack of significant ANOVA results or low predator recoveries. Please note the change in scale for the y-axis.

A significant vegetation type by time period interaction was detected for *Coccinella septempunctata* in 2018 (F = 3.01; d.f. = 8, 135; *p* = 0.0039) (Figure 2a). *Coccinella septempunctata* was detected only during one time period in 2019 (25 April); therefore, analysis was not conducted. In 2018, *C. septempunctata* was collected from all vegetation types (Figure 2a, note change in y-axis scale), but its abundance was variable and relatively low, resulting in no significant differences detected across vegetation types for each time period. This lady beetle species is non-native but is widely distributed across North America [33]. *Coccinella septempunctata* was also detected preying on *M. sorghi* on grain sorghum in the northcentral Great Plains [8]. This lady beetle species has been shown to suppress *M. sorghi* populations below action thresholds in greenhouse studies [34] and was present in sorghum in the early- to mid-sorghum-growing season in our study (Figure 2a). *Coccinella septempunctata* population density has been shown to be greater in areas where grain sorghum and cotton grow within the same farmscape [35]. This may apply in the agroecosystem of our study, where *C. septempunctata* was most abundant throughout the period of cultivated sorghum (March to May) and persisted after sorghum harvest when cotton was still in production (April to August) (Figure 2a).

Variation across time period (but not vegetation type) was detected for *Scymnus* spp. in 2018 (F = 5.14; d.f. = 4, 8; *p* = 0.0007) and the interaction was not significant *(p* > 0.05) in 2018 (Figure 2b). In 2019, *Scynmus* spp. were rarely detected and variable (mean < 0.2 per pot and the SEM was equal to the mean); therefore, analysis was not conducted. *Scymnus* spp. abundance was low, but most abundant at the beginning of the sorghum growing season (Figure 2b). *Scymnus* spp. were abundant in south and central Texas [11], and they are known predators of pests in cotton [36] and corn [37] which are grown in the sorghum production region where this study was conducted.

Variation across time period (F = 14.12; d.f. = 4, 8; *p* < 0.0001) and vegetation type (F = 5.19; d.f. = 2, 8; *p* = 0.0067) was detected for *Cycloneda sanguinea* in 2018, and the interaction was not significant *(p* > 0.05) (Figure 2c). *Cycloneda sanguinea* was rarely detected in 2019 (it was detected in only three pots); therefore, analysis was not conducted. *Cycloneda sanguinea* was most relatively abundant in all vegetation types early during sorghum cultivation and again after harvest (Figure 2c). It was more sporadic during other time periods. Averaging across time points, it was more common in the two non-crop vegetation types than in sorghum (Figure 2c). This species is a main predator in cotton [38]. Cotton is the main rotational crop with sorghum (corn as well where water availability allows) in south Texas through the southcentral Great Plains. Its relatively greater presence early in sorghum production and off-season demonstrated in our study deserves additional study. It may have a special affinity to riparian areas, which positions it to prey on *M. sorghi* during the early period of sorghum cultivation. Given its presence in cotton as well, it appears to be a main aphid predator across the sorghum/cotton agroecosystem.

### 3.3. Melanaphis sorghi Densities

*Melanaphis sorghi* populations grew and exceeded the economic threshold of 40 aphids per leaf used in the region [4] as cultivated sorghum matured in 2018. Parasitoids and predators were present in each of the habitat sites in 2018. In contrast, *M. sorghi* populations initially increased in 2019 but declined though head maturation and remained at a low level in remnant sorghum after harvest (Table 2). These observations coincided with greater numbers of parasitoids and predators collected off-season in the spring and the early in-season of sorghum cultivation in 2019 (Figure 1 and Figure 2).

## 4. Conclusions

These results highlight the potential importance of persistence of natural enemies across vegetation types associated with their ability to suppress *M. sorghi* infestations. The vegetative types of this study are relevant components of the sorghum agroecosystem and were found to harbor natural enemies of *M. sorghi* in- and off-season to varying degrees, depending on the natural enemy taxa. *Apehlinus nigritus* abundance across time and the three vegetation types bodes well for it to have a top-down suppression influence on *M. sorghi* and is consistent with previous information on its response of *M. sorghi* on sorghum [8,11,26,27]. We conclude that the natural enemy species composition and seasonal variation in abundance across vegetation types may be useful indicators of top-down regulatory potential for *M. sorghi*. The quality and quantity of top-down suppression by these enemies deserves additional attention. Simultaneously, data collection of natural enemies and *M. sorghi* in sorghum and non-crop vegetation combined with controlled experimentation designed to authenticate *M. sorghi* suppression are advised. The study highlights key natural enemies of interest for further study.

## Figures and Tables

**Table 1:** Total number of natural enemy taxa (N) collected during the sorghum growing season (in-season) and when sorghum in not in cultivation (off-season). A total of 210 and 90 sentinel pots were placed in-season and off-season, respectively, equally across three vegetation types of the sorghum agroecosystem during the two-year study.

	In-Season			Off-Season		
Natural Enemy	N	% of Total	Cell χ^2^	N	% of Total	Cell χ^2^
Parasitoids						
*Aphelinus nigritus*	332	36.69	6.62	54	5.97	20
*Lysiphlebus testaceipes*	48	5.3	20.4	74	8.18	60
*Syrphophagus aphidivorus*	11	1.22	0.46	1	0.11	1.4
Predators						
*Cycloneda sanguinea*	57	6.3	15.1	70	7.73	45
*Scymnus* spp.	70	7.73	88.6	9	0.99	6
*Coccinella septempunctata*	43	4.75	0.63	8	0.88	1.9
*Hippodamia convergens*	19	2.1	1.63	0	0	4.8
*Coleomegilla maculata*	10	1.1	0.01	3	0.33	0
*Harmonia axyridis*	1	0.11	3.42	6	0.66	10
Coccinellidae larvae	75	8.29	4.81	3	0.33	14
Syrphidae larvae	6	0.66	0.11	1	0.11	0.3
Chrysopidae/Hemerobiidae larvae	4	0.44	0.34	0	0	1

The equality test of proportions indicated disproportionately high rates of recovery in-season (χ^2^ = 142; df = 11; *p ≤* 0.0001), off-season (χ^2^ = 177; df = 11; *p ≤* 0.0001), or both of selected natural enemies across the three vegetative types. Counts are for adults unless otherwise indicated.

**Table 2:** Average number of *M. sorghi* per leaf in Johnson grass and sorghum during grain sorghum cultivation (in-season) and when grain sorghum was not in cultivation (off-season). Note that the earliest that data was collected at the local field site was in April of 2018 and 2019. “--” indicates data not available.

				Johnson Grass	Sorghum	
Season	Julian Date	Year	Date	*M. sorghi* per Leaf	*M. sorghi* per Leaf	Plant Growth Stage
In-Season	110	2018	20-April	6.35	0.63	V6
	152	2018	1-June	7.67	2.6	Flag
	179	2018	28-June	--	7.79	Flower
	193	2018	12-July	--	81.67	Soft Dough
	109	2019	19-April	0.00	0.5	V6
	135	2019	15-May	4.25	--	--
	154	2019	3-June	--	23.5	V6
	197	2019	16-July	--	7.05	Milk
Off-Season	277	2018	4-October	--	6.7	Hard Dough
	277	2019	4-October	0.00	--	--

## Data Availability

The data presented in this study are available on request from the corresponding author.
